# Persistent Coronavirus Disease 2019 (COVID‐19) in Patients With Malignant Lymphoma Treated With Anti‐CD20 Antibodies Who Responded to Ensitrelvir: A Report of Three Cases

**DOI:** 10.1002/rcr2.70804

**Published:** 2026-11-02

**Authors:** Mika Horie, Tamio Okimoto, Yusuke Okada, Masaya Inoue, Shintaro Shimizu, Hirotoki Tsuda, Kojiro Hata, Takae Okuno, Yoshihiro Amano, Mika Nakao, Kazuhisa Nakashima, Megumi Hamaguchi, Takeshi Isobe

**Affiliations:** ^1^ Department of Internal Medicine, Division of Medical Oncology and Respiratory Medicine Shimane University Faculty of Medicine Izumo Shimane Japan; ^2^ Department of Hematology Shimane University Hospital Izumo Japan

**Keywords:** coronavirus disease 2019 (COVID‐19), ensitrelvir, immunocompromised host, malignant lymphoma, persistent COVID‐19

## Abstract

Patients with malignant lymphoma receiving anti‐CD20 antibody therapy are at high risk of persistent coronavirus disease 2019 (COVID‐19), with no established standard treatment. We report three cases of persistent COVID‐19 in patients with follicular lymphoma who responded clinically after switching to ensitrelvir following the failure of remdesivir‐based treatment. Each patient had multiple relapses and an inadequate response to remdesivir but achieved symptom resolution with ensitrelvir. These cases suggest that switching to an antiviral with a different mechanism of action may be an effective therapeutic strategy for immunocompromised patients when initial antiviral therapies fail.

## Graphical abstract

Patients with malignant lymphoma receiving anti‐CD20 antibody therapy are at high risk of persistent coronavirus disease 2019 (COVID‐19). We report three cases of persistent COVID‐19 in patients with follicular lymphoma who responded clinically after switching to ensitrelvir following the failure of remdesivir‐based treatment. Switching to an antiviral with a different mechanism of action may be a therapeutic option for immunocompromised patients when initial antiviral therapies fail.

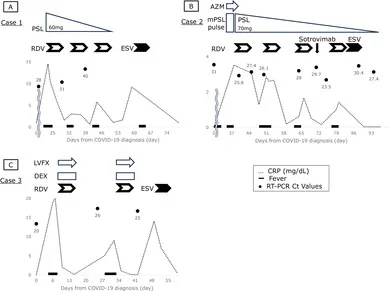

## 1 Introduction

Persistent coronavirus disease 2019 (COVID‐19), characterized by prolonged viral detection and recurrent symptoms [1, 2], poses a therapeutic challenge in immunocompromised patients, particularly those receiving anti‐CD20 antibodies for haematological diseases [3, 4, 5, 6]. In these patients, impaired B‐cell immunity frequently leads to prolonged viral replication and recurrent clinical deterioration.

Ensitrelvir, a SARS‐CoV‐2 3‐chymotrypsin‐like protease (3CLpro) inhibitor, blocks polyprotein cleavage and replication and has been shown to shorten symptom duration and reduce viral load in mild‐to‐moderate COVID‐19 [7, 8]. However, evidence regarding optimal antiviral strategies for remdesivir‐refractory persistent COVID‐19 in patients receiving B‐cell–depleting therapies remains limited, and reports describing the use of ensitrelvir in this setting are still scarce.

Here, we report three patients with follicular lymphoma (FL) who developed remdesivir‐refractory persistent COVID‐19 after anti‐CD20 therapy and subsequently improved after ensitrelvir administration.

## 2 Case Series

### 2.1 Case 1

A 65‐year‐old man with FL, classified as Lugano Stage IVA and low risk according to the Follicular Lymphoma International Prognostic Index (FLIPI), was diagnosed with Grade 2 FL according to the WHO 2008 classification. He initially received six cycles of rituximab, cyclophosphamide, doxorubicin, vincristine, and prednisolone (R‐CHOP). After relapse, he underwent six courses of bendamustine‐rituximab followed by rituximab maintenance therapy. He received the most recent (11th) dose of rituximab 1 month prior to COVID‐19 onset. Recent laboratory tests revealed a soluble interleukin‐2 receptor (sIL‐2R) level of 244 U/mL, lactate dehydrogenase (LDH) of 155 U/L, and beta‐2 microglobulin (B2M) level of 2.1 mg/L. Computed tomography (CT) revealed a partial response. His immunoglobulin levels decreased, with IgG, IgA, and IgM levels at 450, 61, and 9 mg/dL, respectively. His lymphocyte count was 2240 cells/μL. On Day 0 of COVID‐19 onset (March 2024), he developed upper respiratory symptoms and was diagnosed with COVID‐19. Lineages BA.2.86, EG.5, and JN.1 were circulating. On Day 22, he was admitted with fever, anorexia, and hypoxemia. The polymerase chain reaction (PCR) cycle threshold (Ct) value was 28.3, and chest CT revealed faint ground‐glass opacities with interlobular septal thickening. Remdesivir and prednisolone (1 mg/kg, 60 mg) improved symptoms, but relapse followed discontinuation. On Day 63, ensitrelvir was administered during the third relapse, resulting in complete resolution (Figure 1A).

**FIGURE 1:**
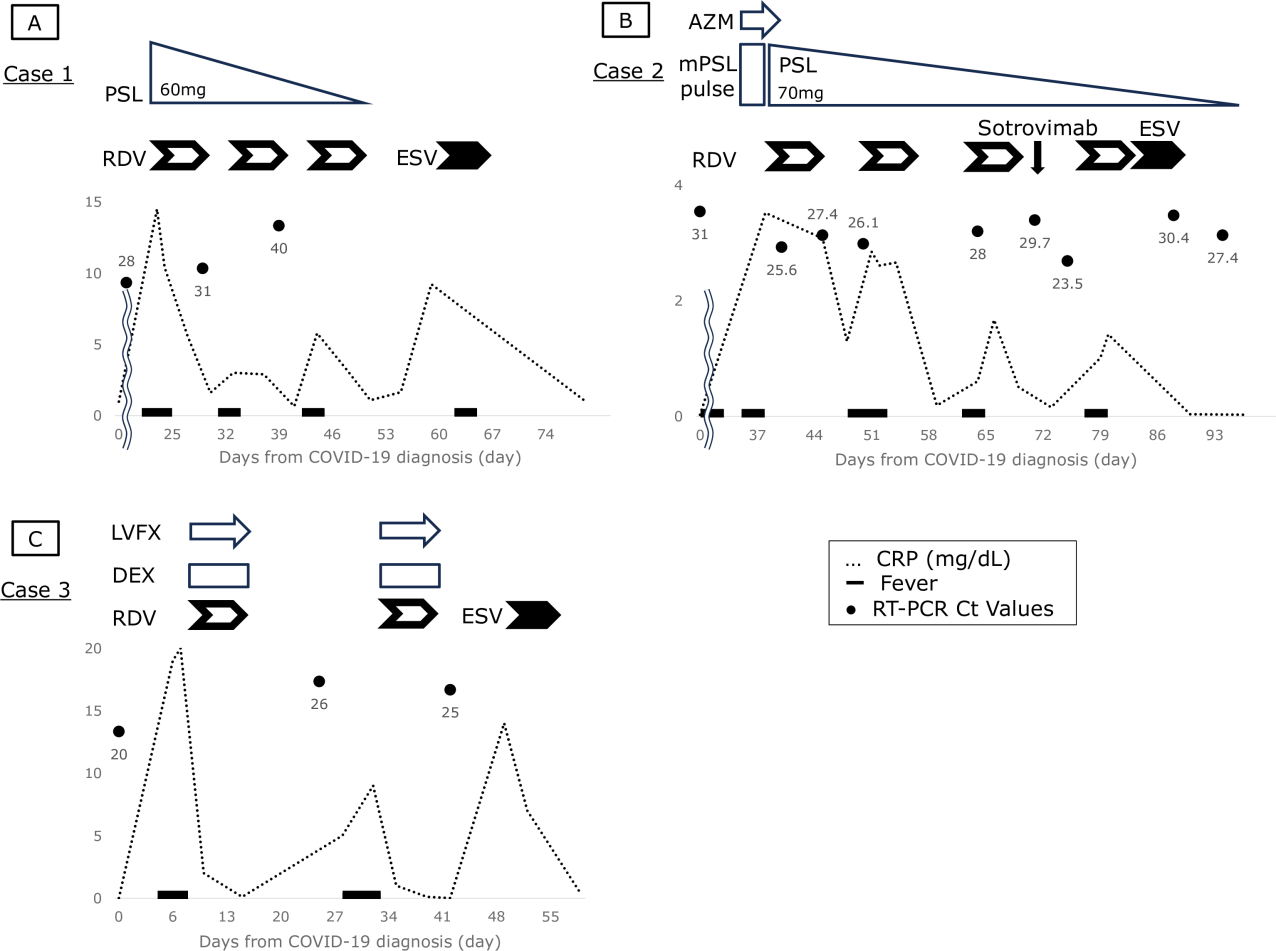
(A–C) Clinical courses of Cases 1, 2, and 3, respectively, showing antiviral treatments and concomitant medications. The dotted lines represent CRP levels, the black horizontal bars indicate periods of fever, and the black circles represent RT‐PCR Ct values. In all three cases, remdesivir‐based treatment was followed by recurrent symptoms despite transient clinical improvement. Subsequent administration of ensitrelvir was associated with clinical improvement without further documented relapses. AZM, azithromycin; CRP, C‐reactive protein; Ct, cycle threshold; DEX, dexamethasone; ESV, ensitrelvir; LVFX, levofloxacin; mPSL pulse, methylprednisolone pulse therapy; PSL, prednisolone; RDV, remdesivir; RT‐PCR, reverse transcription‐polymerase chain reaction.

### 2.2 Case 2

An 81‐year‐old man with FL, classified as having advanced‐stage disease according to the Lugano classification and as high risk according to the FLIPI, was diagnosed with Grade 3A FL according to the WHO 2017 classification. During follow‐up, the disease transformed into Grade 3B FL, and the patient received six cycles of bendamustine‐obinutuzumab (BG), with the most recent dose of BG administered 20 months prior to the onset of COVID‐19. Positron emission tomography‐CT (PET–CT) demonstrated a complete metabolic response (CMR). Recent laboratory tests revealed an sIL‐2R level of 725 U/mL and an LDH level of 240 U/L. His immune status was compromised, with a lymphocyte count of 790 cells/μL and IgG level of 785 mg/dL. On Day 0 (October 2023), he developed upper respiratory symptoms and was diagnosed with COVID‐19. The SARS‐CoV‐2 XBB.1 lineage was circulating. On Day 31, he developed recurrent fever, cough, and respiratory failure. The Ct value was 28, and chest CT showed ground‐glass opacities in the lower lobes. Remdesivir, steroid pulse therapy, and azithromycin improved symptoms transiently, but relapse occurred after discontinuation. On Day 83, ensitrelvir was administered at the third relapse, preventing further recurrences (Figure 1B).

### 2.3 Case 3

A 59‐year‐old man with FL, classified as Lugano Stage IV and intermediate‐risk according to the FLIPI, was diagnosed with Grade 3A FL according to the WHO 2017 classification. Because his condition worsened during follow‐up, he received six cycles of BG and continued obinutuzumab maintenance therapy. He received his most recent (5th) dose of obinutuzumab 1 month before the onset of COVID‐19. PET–CT revealed CMR. Recent laboratory tests revealed an sIL‐2R level of 620 U/mL, LDH level of 281 U/L, and B2M of 2.0 mg/L. His immune status was compromised, with a lymphocyte count of 230 cells/μL, IgG of 544 mg/dL, IgM of 11 mg/dL, IgA of 44 mg/dL, a calculated absolute CD4+ T‐cell count of 50 cells/μL, and a calculated absolute CD19+ B‐cell count of 0.2 cells/μL. On Day 0 (March 2025), the patient developed upper respiratory symptoms and was diagnosed with COVID‐19. Lineages XEC and KP.3 were circulating. On Day 6, he was admitted with persistent fever without respiratory failure. The Ct value was 20, and chest CT showed ground‐glass and granular opacities. Remdesivir, dexamethasone, and levofloxacin produced temporary improvement, but fever and pneumonia recurred twice. During the second relapse (Day 49), ensitrelvir was started, leading to symptom improvement and no further relapse (Figure 1C).

## 3 Discussion

All three patients who received anti‐CD20 antibodies developed remdesivir‐refractory persistent COVID‐19 with repeated clinical relapses; however, they subsequently improved after ensitrelvir treatment. Similar outcomes have been previously reported (Table 1) [9, 10, 11].

**TABLE 1:** The cases of lymphoma patients treated with anti‐CD20 antibodies who had persistent COVID‐19 showed improvement following a switch from prior antiviral agents to ensitrelvir.

No	Age	Sex	Severity	Lymphoma	Anti‐CD20‐Ab		Treatment						
					Drug	Time since last dose	1st	2nd	3rd	4th	5th	6th	7th
1 [9]	76	Male	MII	MCL	RTX	6y	MOL	RDV	RDV	mAb	RDV	mAb	ESV
2 [9]	78	Male	M	DLBCL	RTX	8d	RDV	RDV	mAb	ESV			
3 [10]	62	Female	MI	FL	RTX	15d	MOL	ESV					
4 [11]	84	Female	—	NHL	Unspecified drug		RDV	ESV					
5 [11]	88	Female	—	NHL	Unspecified drug		RDV	ESV					
6*	65	Male	MII	FL	RTX	1 m	RDV	RDV	RDV	ESV			
7*	81	Male	MII	FL	OBI	20 m	RDV	RDV	RDV	mAb	RDV	ESV	
8*	59	Male	MI	FL	OBI	1 m	RDV	RDV	ESV				

*Note:* An asterisk (*) indicates our own cases.

Abbreviations: Anti‐CD20‐Ab, anti‐CD20 antibody therapy; DLBCL, Diffuse large B‐cell lymphoma; ESV, Ensitrelvir; FL, Follicular Lymphoma; M, Mild; mAb, monoclonal antibody; MCL, Mantle Cell Lymphoma; MI, Moderate I; MII, Moderate II; MOL, Molnupiravir; NHL, Non‐Hodgkin lymphoma; OBI, Obinutuzumab; RDV, Remdesivir; RTX, Rituximab.

Persistent COVID‐19 in patients treated with anti‐CD20 antibodies has emerged as a challenging clinical entity because impaired humoral immunity often results in prolonged viral replication and recurrent clinical deterioration. Although several antiviral agents have been used in this setting, the optimal treatment strategy remains unclear, particularly when the initial antiviral therapy fails. Therefore, switching to an antiviral agent with a different mechanism of action may represent a reasonable therapeutic approach.

Ensitrelvir inhibits SARS‐CoV‐2 3CLpro, whereas remdesivir and molnupiravir inhibit RNA‐dependent RNA polymerase. Antivirals with distinct mechanisms of action may therefore be effective in persistent COVID‐19. Furthermore, immunocompromised patients receiving B‐cell‐depleting therapies have poor viral clearance [12]. Ensitrelvir may be particularly beneficial in these patients because it has shown antiviral efficacy in clinical trials [7, 8]. Additionally, SARS‐CoV‐2 3CLpro has been reported to be involved in antagonizing innate immunity and prompting excessive inflammatory responses [13]. A reduction in inflammatory cytokine and chemokine production has been shown to correlate with the antiviral effect of ensitrelvir [14]. Thus, inhibition of SARS‐CoV‐2 3CLpro may also contribute to anti‐inflammatory effects.

However, the potential contribution of the systemic corticosteroid‐induced immunomodulatory effects to the clinical course cannot be entirely ruled out. Nevertheless, in Cases 1 and 2, high‐dose systemic corticosteroids alone did not resolve fever, suggesting that their immunomodulatory effects were insufficient. In all cases, the symptoms subsided after the administration of antiviral drugs, possibly demonstrating the effectiveness of antiviral treatments in persistent COVID‐19.

This report has several limitations, including the small sample size, the absence of genomic and resistance analyses, and limited follow‐up PCR testing due to cost constraints, which restrict the assessment of virological clearance. Additionally, we did not perform a systematic comparison with other patients with FL treated with remdesivir at our institution. Further studies with larger sample sizes are required to clarify the optimal antiviral strategy for persistent COVID‐19 in patients receiving B‐cell–depleting therapies.

These findings suggest that ensitrelvir may represent a potential therapeutic option for persistent COVID‐19 in patients receiving anti‐CD20 therapy. However, further studies are required. Collectively, our cases highlight the potential role of ensitrelvir as a salvage antiviral agent against remdesivir‐refractory persistent COVID‐19 in immunocompromised patients.

## Author Contributions

**Mika Horie:** conceptualization, visualization, writing – original draft. **Tamio Okimoto:** conceptualization, writing – original draft, visualization, supervision. **Yusuke Okada:** writing – review and editing. **Masaya Inoue:** writing – review and editing. **Shintaro Shimizu:** writing – review and editing. **Hirotoki Tsuda:** writing – review and editing. **Kojiro Hata:** writing – review and editing. **Takae Okuno:** writing – review and editing. **Yoshihiro Amano:** writing – review and editing. **Mika Nakao:** writing – review and editing. **Kazuhisa Nakashima:** writing – review and editing. **Megumi Hamaguchi:** writing – review and editing. **Takeshi Isobe:** writing – review and editing.

## Funding

The authors have nothing to report.

## Consent

The authors declare that written informed consent was obtained from all the patients for the publication of this manuscript and accompanying images and attest that the form used to obtain consent from the patients complies with the Journal requirements as outlined in the author guidelines.

## Conflicts of Interest

The authors declare no conflicts of interest.

## Data Availability

The data that support the findings of this study are available from the corresponding author upon reasonable request.
